# Risk Factors and Clinical Predictors of Suicidal Behaviors and Non-Suicidal Self-Injury Among Pediatric Psychiatry Emergency Admissions Pre- and Post-Pandemic: A Retrospective Cohort Study

**DOI:** 10.3390/children12010081

**Published:** 2025-01-11

**Authors:** Roxana Șipoș, Tudor Văidean, Elena Predescu

**Affiliations:** 1 Department of Neuroscience, Psychiatry and Pediatric Psychiatry, “Iuliu Hatieganu” University of Medicine and Pharmacy, 57 Republicii Street, 400489 Cluj-Napoca, Romania; 2 Department of Clinical Psychology and Psychotherapy, Faculty of Psychology and Educational Sciences, Babeş-Bolyai University, 37 Republicii Street, 400015 Cluj-Napoca, Romania

**Keywords:** adolescent, suicidal behavior, non-suicidal self-injury, risk factors, COVID-19 pandemic

## Abstract

Background: Suicidal behavior (SB) and non-suicidal self-injury (NSSI) are significant public health concerns among adolescents. The COVID-19 pandemic may have exacerbated these issues. Methods: This retrospective cohort study analyzed data from 341 adolescents (aged 6–18 years) presenting to a Romanian pediatric psychiatry emergency department during the years 2019 (pre-pandemic) and 2022 (post-pandemic). All participants underwent a thorough psychiatric assessment, and, together with their caregivers, were questioned on a wide range of potentially relevant issues, such as family, social, school, and life history factors. Logistic regression and random forest models were used to identify predictors of SB and NSSI. Results: SB was significantly predicted in regression models based on a prior suicidal ideation (OR = 68.410; *p* < 0.001), having a parent living abroad (OR = 11.438; *p* = 0.020), depression (OR = 6.803; *p* < 0.001), and conflicts with peers (OR = 0.325, *p* = 0.042), teachers (OR = 0.119, *p* = 0.024), or both (OR = 0.166, *p* = 0.012). The random forest model featured a slightly different order of the main predictors and highlighted the importance of additional predictors, such as prior suicide attempts, gender, and past non-suicidal self-injury. NSSI was mainly predicted by a history of self-harm (OR = 52.437; *p* < 0.001), the number of comorbid psychiatric disorders (OR = 1.709; *p* = 0.003), and conduct disorder (OR = 0.184; *p* < 0.001), to which are added, according to random forest models, new predictors, such as borderline personality disorder, suicidal ideation, and school performance. Post-pandemic increases were observed in depression, suicidal ideation, and possible psycho-traumatic negative life event exposure. Conclusions: This study underscores the complex interplay of individual, familial, and societal factors influencing adolescent self-harm. Comprehensive interventions are needed, with early intervention crucial for those with a history of self-harm. Further research using prospective designs is recommended.

## 1. Introduction

Non-suicidal self-injury (NSSI) involves deliberate self-harm without suicidal intent and is often employed as a maladaptive coping mechanism for emotional distress [1,2]. NSSI is most prevalent during adolescence, a developmental stage marked by heightened emotional vulnerability and risk-taking behaviors [3]. Prevalence rates of NSSI range from 15% to 25% in community samples, rising to as high as 60% among adolescents in clinical settings, such as emergency departments [4,5]. Research indicates that children and adolescents with psychiatric disorders frequently engage in NSSI and suicide attempts and experience suicidal ideation, underscoring the severity of these behaviors in clinical samples [6]. Despite its non-suicidal intent, NSSI is a robust predictor of future suicidal behavior (SB), emphasizing the critical need for targeted interventions in high-risk populations [7,8,9]. However, studies show that empirical support for such interventions for children and adolescents is so far extremely low [10].

Suicidal behavior (SB), encompassing suicidal ideation, attempts, and completed suicide, remains one of the leading causes of mortality among adolescents worldwide [11]. Unlike NSSI, SB is driven by an explicit intent to end one’s life, often stemming from a combination of individual vulnerabilities (e.g., depression, hopelessness), familial stressors, and societal pressures [12]. Shared predictors, such as emotional dysregulation and adverse childhood experiences, complicate the distinction between NSSI and SB, yet research underscores critical differences in their etiology. For instance, while both behaviors are associated with depression and trauma, SB uniquely correlates with impulsivity, hopelessness, and a history of previous suicide attempts [13,14]. Understanding the nuanced predictors of SB and its interplay with NSSI is essential for early identification and prevention.

The COVID-19 pandemic has significantly amplified mental health concerns among adolescents. Pandemic-related stressors—including social isolation, disrupted routines, increased family conflict, and limited access to mental health services—have been linked to a surge in psychological distress and maladaptive behaviors, including NSSI and SB [15,16]. Recent studies indicate that adolescents faced heightened emotional dysregulation during this period, reflecting the pandemic’s exacerbation of pre-existing vulnerabilities, such as depression, anxiety, and adverse childhood experiences [17,18]. Additionally, alarming trends in hospital admissions related to deliberate self-poisoning and substance use among Romanian adolescents have emerged, reflecting the broader mental health challenges during this time [19]. At the same time, protective factors, such as social support, peer networks, and school-based interventions, were significantly reduced, further elevating the risk of self-harm behaviors [20]. These findings emphasize the need to explore the pandemic’s varied impacts on NSSI and SB, particularly within high-risk clinical settings.

Despite the growing body of literature, significant research gaps remain in understanding the specific predictors of NSSI and SB across diverse populations and contexts. Many studies focus on community samples, leaving clinical populations—such as adolescents presenting to emergency departments—underexplored [5,21]. Moreover, much of the research has been conducted in Western countries, limiting the generalizability of findings to other cultural and healthcare contexts. For example, Romania, a country with limited mental health resources for adolescents, remains underrepresented in global studies on self-harm and suicide [22]. Understanding predictors of NSSI and SB within Romanian pediatric psychiatry emergency settings is crucial for tailoring culturally sensitive interventions.

In view of these aspects, the main aim of the present study is to investigate the risk factors and clinical predictors of NSSI and SB among adolescents presenting to a Romanian pediatric psychiatry emergency department before and after the COVID-19 pandemic. More specifically, we aim to identify those factors that determine that only a fraction of patients with mental disorders severe enough to present in emergency conditions resort to such behaviors. To this end, we used a retrospective cohort design in which we applied logistic regression and random forest modeling to identify the potential predictors. The underlying hypothesis is that by combining these two statistical procedures we could identify not only factors that already benefit from empirical support (such as certain psychiatric disorders) but also factors that have not been discussed in the literature so far, such as aspects related to the family, social, school, or life history background of patients. Another assumption is that the COVID-19 pandemic could have influenced the dynamics of the factors influencing NSSI and SB, by changing the weights of certain variables or even by introducing some new elements. By exploring the pandemic’s impact on these predictors, this research aims to inform the development of targeted prevention and intervention strategies for high-risk populations, ultimately contributing to a better understanding of the mechanisms linking NSSI and SB.

## 2. Materials and Methods

### 2.1. Participant Selection

This retrospective cohort study analyzed data from 341 children and adolescents aged 6 to 18 years who were admitted as emergencies to the Pediatric Psychiatry Clinic in Cluj-Napoca during 2019 and 2022. These years were selected to enable a comparative analysis of psychiatric emergencies before (2019) and after (2022) the COVID-19 pandemic. All patients meeting the inclusion criteria were included, with those who did not engage in non-suicidal self-injury (NSSI) or suicidal behavior (SB) serving as the control group.

Inclusion criteria required participants to be between 6 and 18 years of age, admitted for an emergency psychiatric evaluation or treatment, and to have provided informed consent for the use of their medical data for research purposes. Consent was obtained from patients and from their parents or legal guardians. Patients who exclusively utilized outpatient services, declined consent, or had incomplete medical records were excluded.

Psychiatric diagnoses were assigned according to the International Classification of Diseases, 10th Revision (ICD-10), based on comprehensive clinical evaluations conducted by trained child psychiatrists. The study adhered to the principles of the Declaration of Helsinki and received approval from the Emergency Clinical Hospital for Children Cluj-Napoca Clinical Trials Quality Assurance Commission (Approval No. 80/14.12.2020).

### 2.2. Data Collection

Data were retrospectively extracted from medical records by two independent raters, with discrepancies resolved through a consensus. The dataset included socio-demographic information, family and personal history, clinical characteristics, school-related variables, and self-harm behaviors.

Socio-demographic and clinical information was primarily based on self-reported or parent-reported data documented during clinical evaluations. Reports included details on the socio-economic status, family structure and dynamics, and school-related factors, such as academic performance, bullying, peer conflicts, and disciplinary issues. Clinician observations during emergency admissions supplemented self- or parent-reported accounts where possible.

Psychiatric diagnoses were classified according to ICD-10 and included affective disorders (e.g., depressive episodes, bipolar disorder), anxiety and stress-related disorders (e.g., generalized anxiety disorder, post-traumatic stress disorder [PTSD]), conduct and emotional disorders (e.g., oppositional defiant disorder, adjustment disorder), neurodevelopmental disorders (e.g., attention deficit hyperactivity disorder [ADHD], autism spectrum disorder), substance use disorders, psychotic disorders, and other categories (e.g., eating disorders or somatoform disorders). Personality disorder traits were also recorded when present.

In addition to establishing the psychiatric diagnosis, the assessment of all participants involved asking predetermined questions investigating their family, school, and life context. Information on chronic somatic illnesses, prior psychiatric treatments (e.g., psychotherapy, pharmacological interventions), and potentially psycho-traumatizing negative life events (i.e., parental absence, death of a close person, school failure, history of suicide in the family, serious interpersonal conflicts, severe illness in the family, a history of accidents, or any other event that the patient may have considered to be psycho-traumatizing) was collected. Family-related variables included the parental age, marital status (married, single, divorced/separated, or widowed), employment status, education level, family conflicts, and family history of psychiatric disorders (e.g., depression, anxiety, schizophrenia, bipolar disorder, or substance use disorders).

Self-harm behaviors, including non-suicidal self-injury (NSSI) and suicidal behavior (SB, encompassing suicidal ideation and attempts), were also primarily reported by participants or their parents during emergency consultations. The frequency, method, and circumstances of these behaviors were documented in the medical records by attending clinicians.

### 2.3. Statistical Analysis

Statistical analyses were conducted using SPSS software (version 17.0) and R (version 4.3.3 for MacOS) alongside R Studio (version 2023.12.1+402) for advanced statistical modeling. Descriptive statistics summarized continuous variables as means and standard deviations, while categorical variables were presented as frequencies and percentages. Analyses were stratified by year (2019 vs. 2022) and by self-harm category, which included a control group, suicidal behaviors (SBs), and non-suicidal self-injury (NSSI).

Comparative analyses employed independent t-tests for continuous variables (e.g., age) and chi-square tests for categorical variables (e.g., gender, diagnosis, school-related conflicts). Given the large number of variables under consideration, Bonferroni corrections or other procedures to handle multiple comparisons were not performed, since such approaches tend to be particularly conservative in such situations and, in attempting to reduce Type I errors, there would have been a high risk of sacrificing statistically significant findings. Instead, to achieve the predictions of interest, we used two different statistical procedures that effectively control for covariates without, however, overdoing it in terms of stringency, and we reported 95% confidence intervals to provide a clearer understanding of the data without stringent multiple correction adjustments.

First, logistic regression models were developed to predict suicidal behavior and NSSI based on variables, including sex, environment, psychiatric diagnosis, and school/family-related conflicts. Two logistic regression models were created for each outcome variable to assess the predictive power of different sets of predictors. Categorical predictors were dummy-coded, and ordinal predictors (e.g., socioeconomic status) were treated as ordinal variables. Supplementary Table S1 displays the predictor variables used in each of the two regression models.

To address potential multicollinearity among predictor variables and to account for complex, non-linear relationships, random forest (RF) models were employed alongside logistic regression. The RF models comprised 2000 decision trees, with hyperparameters (e.g., mtry, sample.fraction, and min.node.size) optimized through a grid search to minimize the root mean squared error (RMSE). Final models utilized mtry = 8, sample.fraction = 0.80, and min.node.size = 5. Sampling without replacement was applied to avoid selection bias [23]. The random forest algorithm is particularly beneficial for managing health-related data, as it can capture intricate interactions among variables and provide insights into complex relationships, making it a valuable tool in the analysis of health data [24]. Statistical significance was set at *p* < 0.05.

## 3. Results

### 3.1. Characteristics of the Sample

The study sample included 341 participants, consisting of 200 females and 141 males, aged between 6 and 17 years, with a mean age of 14.57 years (SD = 2.25). The majority of participants (*n* = 214) were from urban areas. Most of the participants (*n* = 190) came from stable family environments, although many reported experiencing socioeconomic challenges (*n* = 190). A significant portion of the sample (71.85%) indicated a history of familial conflict, while 58.65% reported having at least one family member diagnosed with a psychiatric disorder, with substance use disorders (SUDs), particularly tobacco and alcohol use, being the most common (33.13%). Additionally, 14.95% (*n* = 51) reported a family history of somatic conditions, whereas only 8 participants (2.35%) indicated a neurological disorder in their family history.

A substantial proportion of participants (55.13%, *n* = 188) reported having experienced at least one significant potentially traumatic negative life event, with parental separation/divorce being the most frequently reported (*n* = 64). Regarding educational experiences, while most participants (*n* = 299) were enrolled in school, many reported challenges, including conflicts (*n* = 234) and poor academic performance (*n* = 210). However, only a small proportion (16.1%, *n* = 55) considered family and/or peer conflicts to be severe enough to be categorized as psycho-traumatic experiences.

In terms of psychiatric diagnoses, the most prevalent conditions included depression (41.93%, *n* = 143), conduct disorder (CD; *n* = 135), substance use disorder (SUD; *n* = 134), and attention-deficit/hyperactivity disorder (ADHD; *n* = 131). On average, participants had 3.13 comorbid conditions (SD = 1.38), with some individuals having as many as eight distinct diagnoses. At the time of the study, 23.16% of participants were receiving psychotherapy, while 21.99% reported not using any form of psychotropic medication. All characteristics of the whole sample are presented in Supplementary Table S2.

The study also compared data collected before (2019) and after (2022) the COVID-19 pandemic. Of the participants, 39.29% were assigned to the control group, while 164 participants exhibited suicidal behaviors, including suicidal ideation (*n* = 157) and suicide attempts (*n* = 81). Additionally, 119 participants had engaged in non-suicidal self-injury (NSSI). In total, 163 participants had a history of suicidal ideation, 74 had attempted suicide, and 158 had engaged in NSSI at some point in their lives. Table 1 and Table 2 provide detailed comparisons of these characteristics between the pre-pandemic (2019) and post-pandemic (2022) periods.

Cutting and scratching were the most prevalent self-harm methods, increasing significantly from 62.85% pre-pandemic to 79.59% post-pandemic, χ^2^ (1, *N* = 119) = 3.826, *p* = 0.050, OR = 0.434, 95% CI [0.186, 1.012]. Table 3 presents these data.

### 3.2. Stratification by Assessment Period and Group Comparisons

When stratifying the sample based on the assessment period, several significant differences emerged between the pre- and post-pandemic groups. Participants in the post-pandemic sample had significantly higher mean ages (t(339) = 2.694, *p* = 0.007), *d* = −0.295, 95% CI [−0.510, −0.079], higher depression rates (χ^2^(1, *N* = 341) = 4.297, *p* = 0.038), OR = 1.583, 95% CI [1.024, 2.446], and a greater prevalence of family conflict (χ^2^(1, *N* = 341) = 5.206, *p* = 0.023), OR = 1.769, 95% CI [1.081, 2.897]. They also reported more severe conflicts with parents and/or friends (χ^2^(1, *N* = 341) = 4.698, *p* = 0.030), OR = 1.893, 95% CI [1.057, 3.390], higher rates of suicidal ideation (χ^2^(1, *N* = 341) = 9.869, *p* = 0.002), OR = 2.002, 95% CI [1.295, 3.093], and increased suicidal behavior (χ^2^(1, *N* = 341) = 7.249, *p* = 0.007), OR = 1.809, 95% CI [1.173, 2.790]. Notably, past suicidal ideation rates and prior reports of suicidal behavior did not significantly differ between the two samples.

Educational outcomes also varied significantly between the pre- and post-pandemic groups (χ^2^(3, *N* = 341) = 10.573, *p* = 0.014). More students in the post-pandemic period reported good academic performance (χ^2^(1, *N* = 341) = 8.855, *p* = 0.003), OR = 2.763, 95% CI [1.387, 5.505]. Conversely, post-pandemic participants had significantly lower rates of oppositional defiant disorder (χ^2^(1, *N* = 341) = 5.672, *p* = 0.017), OR = 0.192, 95% CI [0.043, 0.865], sleep disorders (χ^2^(1, *N* = 341) = 10.333, *p* = 0.001), OR = 0.198, 95% CI [0.067, 0.584], and academic conflicts (χ^2^(1, *N* = 341) = 13.993, *p* < 0.001), OR = 0.414, 95% CI [0.260, 0.661].

#### 3.2.1. Suicidal Behavior Group vs. Control Group

When comparing participants in the suicidal behavior group to those in the control group, several significant differences were observed. Participants in the suicidal behavior group were older (t(296) = −2.642, *p* = 0.009), *d* = 0.308, 95% CI [0.078, 0.537] and more likely to be female (χ^2^(1, *N* = 298) = 23.640, *p* < 0.001), OR = 3.264, 95% CI [2.010, 5.299]. They exhibited significantly higher rates of depression (χ^2^(1, *N* = 298) = 77.798, *p* < 0.001), OR = 10.395, 95% CI [5.958, 18.136], anxiety disorders (χ^2^(1, *N* = 298) = 10.100, *p* = 0.001), OR = 2.276, 95% CI [1.363, 3.801], post-traumatic stress disorder (PTSD; χ^2^(1, *N* = 298) = 10.216, *p* = 0.001), OR = 22.049, 95% CI [1.293, 375.954], and borderline personality disorder traits (χ^2^(1, *N* = 298) = 18.642, *p* < 0.001), OR = 4.547, 95% CI [2.189, 9.445]. They also reported more potentially traumatic negative life events (χ^2^(1, *N* = 298) = 12.046, *p* < 0.001), OR = 2.271, 95% CI [1.424, 3.622], higher school attendance (χ^2^(1, *N* = 298) = 7.978, *p* = 0.005), OR = 2.882, 95% CI [1.349, 6.156], and greater participation in regular psychotherapy sessions (χ^2^(1, *N* = 298) = 4.421, *p* = 0.036), OR = 1.825, 95% CI [1.037, 3.211].

In contrast, conduct disorder (χ^2^(1, *N* = 298) = 10.288, *p* = 0.001), OR = 0.464, 95% CI [0.290, 0.744], and sleep disorders (χ^2^(1, *N* = 298) = 7.814, *p* = 0.005), OR = 0.249, 95% CI [0.088, 0.706], were significantly less common in the suicidal behavior group. Participants in this group also had significantly higher frequencies of past suicidal ideation (χ^2^(1, *N* = 298) = 149.222, *p* < 0.001), OR = 36.931, 95% CI [18.892, 72.193], suicide attempts (χ^2^(1, *N* = 298) = 90.887, *p* < 0.001), OR = 262.557, 95% CI [16.067, 4290.431], and past non-suicidal self-injury (NSSI; χ^2^(1, *N* = 298) = 71.286, *p* < 0.001), OR = 10.332, 95% CI [5.740, 18.598].

#### 3.2.2. NSSI Group vs. Control Group

Participants in the NSSI group also exhibited significant differences compared to the control group. They had a higher proportion of females (χ^2^(1, *N* = 253) = 11.101, *p* < 0.001), OR = 2.364, 95% CI [1.419, 3.937], and a greater number of comorbid conditions (t(251) = 3.512, *p* < 0.001), *d* = 0.442, 95% CI [0.192, 0.692]. Depression (χ^2^(1, *N* = 253) = 34.483, *p* < 0.001), OR = 5.249, 95% CI [2.953, 9.332], PTSD (χ^2^(1, *N* = 253) = 9.303, *p* = 0.002), OR = 20.507, 95% CI [1.171, 359.224], borderline personality disorder traits (χ^2^(1, *N* = 253) = 25.855, *p* < 0.001), OR = 6.045, 95% CI [2.857, 12.790], family conflicts (χ^2^(1, *N* = 253) = 9.008, *p* = 0.003), OR = 2.439, 95% CI [1.351, 4.405], and participation in psychotherapy sessions (χ^2^(1, *N* = 253) = 7.513, *p* = 0.006), OR = 2.264, 95% CI [1.253, 4.091], were all more common in the NSSI group. However, conduct disorder was significantly less prevalent in this group (χ^2^(1, *N* = 253) = 8.474, *p* = 0.004), OR = 0.469, 95% CI [0.281, 0.784].

Additionally, participants in the NSSI group reported significantly higher frequencies of past suicidal ideation (χ^2^(1, *N* = 253) = 91.060, *p* < 0.001), OR = 18.291, 95% CI [9.413, 35.543], past self-harm behaviors (χ^2^(1, *N* = 253) = 150.737, *p* < 0.001), OR = 63.273, 95% CI [28.585, 140.052], past suicide attempts (χ^2^(1, *N* = 253) = 27.182, *p* < 0.001), OR = 137.038, 95% CI [8.312, 2259.437], and overall suicidal behavior (χ^2^(1, *N* = 253) = 122.326, *p* < 0.001), OR = 473.069, 95% CI [28.716, 7793.356].

The mean number of past suicide attempts was 0.640 (SD = 0.798) in the suicidal behavior group, with a maximum of four attempts, compared to 0.504 (SD = 0.780) among participants in the NSSI group. Table 4 summarizes demographic characteristics of the suicidal behavior, NSSI, and control groups, while Table 5 details their primary diagnoses.

### 3.3. Logistic Regressions

#### 3.3.1. Predicting Suicidal Behavior (SB)

Logistic regression Model 1 significantly predicted suicidal behavior, (χ^2^(312) = 236.661, *p* < 0.001), accounting for 57.1% of the variance in the sample (Tjur R^2^ = 0.571). Details of the regression coefficients and model fit statistics are provided in Supplementary Table S3. Three variables emerged as significant predictors of suicidal behavior. Depression was associated with an increased risk, with a regression coefficient (β) of 1.740 and an odds ratio (OR) of 5.700 (*p* < 0.001). A history of suicidal ideation was the strongest predictor, β = 3.856, OR = 47.261, *p* < 0.001, indicating that individuals with such a history were approximately 47 times more likely to exhibit suicidal behavior. In contrast, school-related conflicts showed a negative association, β = −1.148, OR = 0.317, *p* = 0.023, suggesting that the presence of school-related conflicts was linked to a reduction in the risk of suicidal behavior by approximately 68%.

Overall, Model 1 demonstrated robust performance, with an accuracy of 84.5%, a sensitivity of 83.5%, a specificity of 85.3%, and an area under the curve (AUC) of 0.928. Multicollinearity, assessed using the Variance Inflation Factor (VIF), was within acceptable limits, with a maximum VIF of 2.297, indicating no significant redundancy among the predictors.

Incorporating additional predictors in Model 2 (χ^2^(298) = 252.910, *p* < 0.001) significantly enhanced its predictive power for self-harm behaviors. The explained variance increased to 59.9% (Tjur R^2^ = 0.599), alongside improvements in the overall accuracy (86.5%), AUC (0.937), sensitivity (86.0%), and specificity (87.0%). Multicollinearity remained moderate, with a maximum Variance Inflation Factor (VIF) below 5, ensuring reliable estimates. Depression (β = 1.917, OR = 6.803, *p* < 0.001) and prior suicidal ideation (β = 4.226, OR = 68.410, *p* < 0.001) remained significant predictors, with their effects amplified in this model.

Further analysis of school-related conflicts, which were previously significant in Model 1, revealed additional insights. Conflicts with peers (β = −1.125, OR = 0.325, *p* = 0.042), teachers (β = −2.130, OR = 0.119, *p* = 0.024), or both (β = −1.797, OR = 0.166, *p* = 0.012) were significantly associated with a reduced risk of suicidal behavior. Notably, Model 2 also identified a new significant risk factor, having a parent living abroad (β = 2.437, OR = 11.438, *p* = 0.020), suggesting that this factor may uniquely contribute to the likelihood of self-harm behaviors. These expanded findings, presented in Supplementary Table S4, underscore the importance of examining diverse contextual factors when predicting suicidal behavior.

#### 3.3.2. Predicting NSSI

The logistic regression Model 1 significantly predicted non-suicidal self-injury (NSSI) behaviors (χ^2^(312) = 197.244, *p* < 0.001), explaining 51.0% of the variance (Tjur R^2^ = 0.510). Several key predictors emerged as significant in the model. Conduct disorder was associated with a significantly reduced risk of NSSI (β = −1.468, OR = 0.230, *p* = 0.001), indicating a protective effect. Conversely, a higher number of comorbid diagnoses (β = 0.554, OR = 1.741, *p* = 0.001) and, most notably, a history of self-harm (β = 3.739, OR = 42.054, *p* < 0.001) were strongly associated with an increased risk. Individuals with a history of self-harm demonstrated a striking 42-fold higher likelihood of engaging in future NSSI.

Model 1 demonstrated strong predictive performance, achieving an accuracy of 84.5%, an AUC of 0.911, sensitivity of 80.7%, and specificity of 86.5%. Multicollinearity was assessed and found to be moderate, indicating stable parameter estimates. Detailed regression coefficients and model fit statistics are presented in Supplementary Table S5.

The addition of predictors in Model 2 (χ^2^(298) = 207.099, *p* < 0.001) slightly enhanced the predictive capacity for NSSI, increasing the explained variance to 52.8% (Tjur R^2^ = 0.528). Model 2 also demonstrated modest gains in accuracy (85.0%), the AUC (0.920), and specificity (87.4%), while maintaining similar sensitivity to Model 1. Consistent with the findings from Model 1, conduct disorder remained a significant negative predictor of NSSI (β = −1.691, OR = 0.184, *p* < 0.001), reinforcing its protective association. In contrast, higher comorbidity (β = 0.536, OR = 1.709; *p* = 0.003) and a history of self-harm (β = 3.960, OR = 52.437; *p* < 0.001) emerged as strong positive predictors, with the latter suggesting a more than 52-fold increase in the likelihood of future NSSI. These results underscore the pivotal role of self-harm history in identifying individuals at a heightened risk. Comprehensive regression coefficients and model fit statistics are provided in Supplementary Table S6.

### 3.4. Random Forest Regressions

#### 3.4.1. Predicting Suicidal Behavior

The random forest model explained 47.0% of the variance in suicidal behavior (R^2^ = 0.470), slightly less than the logistic regression models, with a prediction error of 13.26%. Both impurity- and permutation-based importance measures consistently identified prior suicidal ideation (impurity: 16.7; permutation: 0.129) and depression (impurity: 7.66; permutation: 0.039) as the top predictors. These variables had also been significant in the logistic regression models, with their ranking remaining consistent across methods.

Interestingly, prior suicide attempts, which were not statistically significant in the logistic regression models (e.g., Model 2: β = −0.054, OR = 0.947, *p* = 0.919), ranked as the third most important predictor in the random forest model (permutation importance: 0.00931). Additional influential predictors included female sex (permutation importance: 0.00591) and a history of NSSI (permutation importance: 0.00572), both of which demonstrated relatively stable predictive power.

In contrast, the three facets of school-related conflict—significant in the logistic regression models—contributed minimally to the random forest model’s predictive accuracy. Among these, conflicts with both teachers and peers ranked 11th (permutation importance: 0.00179), followed by conflicts with peers alone (23rd; permutation importance: 0.000277), while conflicts with teachers alone ranked near the bottom with a negative permutation importance score (−0.000557).

The timing of assessment, categorized as pre- versus post-pandemic, was the ninth most important variable, with a permutation importance score of 0.00234. This variable demonstrated greater relevance than certain potentially psycho-traumatizing negative life events, such as severe conflict (ranked 10th; 0.00190), parental divorce (ranked 13th; 0.00173), or having a parent residing abroad. Notably, while parental absence had been statistically significant in the second logistic regression model, it ranked 38th in the random forest model, with a negative permutation importance score (−0.000332).

**Figure 1:**
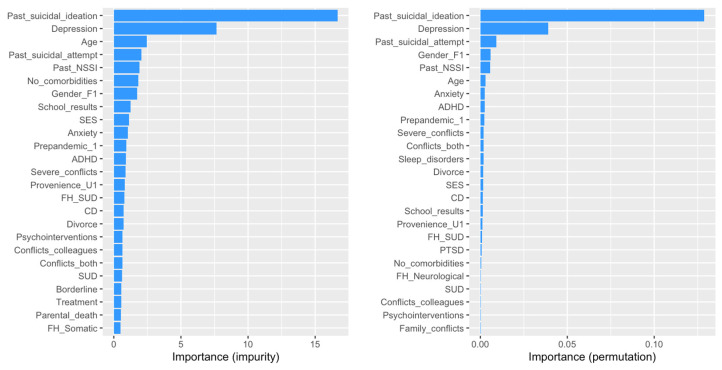
Importance of predictors for suicidal behavior.

A complete breakdown of variable importance scores is available in Supplementary Table S7 and visually depicted in Figure 1. These findings highlight the strengths of the random forest approach in identifying the nuanced contributions of predictors, offering a complementary perspective to the logistic regression results.

#### 3.4.2. Predicting NSSI

The random forest model for predicting non-suicidal self-injury (NSSI) explained 40.3% of the variance (R^2^ = 0.403; prediction error = 13.59%), which was lower than the variance explained by the logistic regression models. Consistent with the logistic regression results, a history of NSSI was the most important predictor, with both impurity (16.9) and permutation (0.136) importance scores highlighting its significance. Borderline personality disorder traits, though showing a relatively low permutation importance score (0.0107), emerged as the second most influential predictor in the random forest model. This contrasts the logistic regression models, where borderline personality disorder traits were not significantly associated with NSSI (e.g., Model 2: β = 0.100, OR = 1.105, *p* = 0.824).

The number of comorbidities, which was a significant predictor in the logistic regression models, showed lower importance in the random forest model (permutation importance: 0.00895), similar to prior suicidal ideation (permutation importance: 0.00877). Other notable predictors in the random forest model included academic performance (0.00569), conduct disorder (0.00544), and anxiety disorders (0.00282).

The timing of presentation, categorized as pre- versus post-pandemic, was ranked eighth in permutation importance (0.00175), surpassing variables, such as past suicide attempts (0.00150; ranked 10th), family conflicts (0.00115; ranked 12th), and other potentially traumatic life events. Among these events, the experience of having a parent living abroad had the lowest permutation importance score (0.000518), ranking 17th.

**Figure 2:**
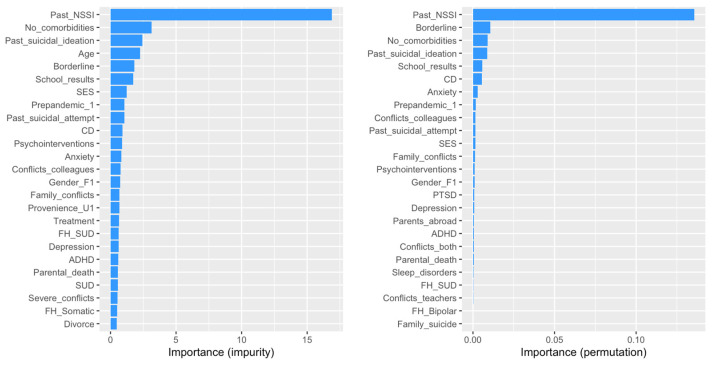
Importance of predictors for NSSI.

These results are detailed in Supplementary Table S8 and illustrated in Figure 2.

## 4. Discussion

This retrospective cohort study investigated the risk factors and clinical predictors associated with suicidal behavior (SB) and non-suicidal self-injury (NSSI) among pediatric psychiatry emergency admissions, contrasting data from the pre-pandemic year of 2019 with the post-pandemic year of 2022. Our comprehensive analysis elucidates several significant associations that enhance and refine the existing literature surrounding adolescent self-harm in clinical environments.

### 4.1. Prevalence and Characteristics of Self-Harm

Our sample comprised a notable majority of females (58.65%) with a mean age of 14.57 years (SD = 2.25), mainly hailing from urban settings (62.75%). While many participants came from ostensibly stable family units, a considerable subset (71.85%) reported experiencing familial conflicts. Furthermore, a significant proportion (58.65%) identified at least one family member with a psychiatric disorder, predominantly substance use disorders (33.13%). These findings echo existing research highlighting the familial transmission of psychopathology and the interplay between emotional and behavioral dysfunctions across generations [25,26,27]. Additionally, we observed that 14.95% of participants reported a family history of somatic conditions, hinting at a possible nexus between mental and physical health vulnerabilities within families. The prevalence of exposure to at least one potentially traumatic negative life event (55.13%), with parental separation or divorce being the most reported (*n* = 64), underscores the importance of considering family dynamics in the assessment and treatment of self-harm behaviors in adolescents [28].

The high prevalence of self-harming behaviors (both SB and NSSI) discovered within our sample aligns with the growing body of evidence documenting the significant burden of these behaviors among adolescents [4,5]. Previous studies have similarly demonstrated elevated rates of self-harm and suicidal ideation among adolescents, particularly in clinical settings [29,30]. Notably, we observed a significant increase in self-harm methods, specifically cutting and scratching, in the post-pandemic period (χ^2^ = 3.826, *p* = 0.050). This escalation reflects broader trends reported in the literature, which raised concerns about heightened rates of self-harm during and following the COVID-19 pandemic [15,16,18,31].

The trend of increased self-harm during the pandemic has been substantiated by several studies, indicating that psychological stressors, such as isolation, uncertainty, and changes in routine, contributed to heightened distress among adolescents [20,32]. Pertinently, medication ingestion was identified as the most common method used in suicide attempts among our sample. This finding is consistent with previous research suggesting that adolescents increasingly utilize pharmaceuticals as a method of self-harm [33,34,35]. Many studies have reported that the accessibility of medications, coupled with emotional distress, contributes to the prevalence of such attempts among youth [36,37].

### 4.2. Risk and Protective Factors

Our comparative analyses yielded several significant risk factors associated with both suicidal behavior (SB) and non-suicidal self-injury (NSSI) among adolescents admitted to psychiatric emergency services.

**Prevalence of Psychiatric Disorders.** Individuals within the SB group demonstrated markedly higher rates of depressive disorders, anxiety disorders, post-traumatic stress disorder (PTSD), borderline personality disorder traits (BPDs), and a history of potentially traumatic negative life events, all with statistical significance (*p* < 0.05). This finding corroborates a substantial body of literature that has consistently linked psychiatric disorders with suicidal behaviors. For instance, Barrocas et al. (2015) and Deutz et al. (2016) highlighted the strong association between major depressive disorder and increased suicide risk among adolescents [38,39]. Fang et al. (2024) also concluded that anxiety disorders serve as robust risk factors for both suicidal ideation and attempts, emphasizing the multifaceted nature of these conditions and their impact on youth [40].

Furthermore, PTSD has been recognized as a significant predictor of suicidal behaviors [41]. Adolescents with PTSD often grapple with intrusive memories and heightened emotional distress, which can contribute to self-harm and suicidal ideation [42]. In our analysis, we found that a history of suicidal ideation and attempts, as well as NSSI, were strongly correlated with current suicidal behaviors. This aligns with previous findings by Ribeiro (2016), who pointed out that such a history should be considered a critical indicator of future suicide risk [8].

**Gender Differences in Suicidal Behavior.** Notably, our study found that females were significantly overrepresented in the SB group (χ^2^ = 23.640, *p* < 0.001). This finding is consistent with established gender differences in suicidal behavior, where studies indicate that adolescent girls tend to exhibit higher rates of suicidal ideation and attempts compared to their male counterparts [43]. Researchers have posited that societal norms, emotional expression differences, and coping styles may contribute to these disparities [44].

**Academic Conflicts and Reporting Bias.** Interestingly, lower rates of academic conflicts were reported in the post-pandemic period, which warrants further investigation [45]. This unexpected finding may reflect a reporting bias, as adolescents may have different thresholds for reporting academic distress after experiencing the unique challenges and disruptions brought on by the pandemic [15]. Alternatively, the complex interplay of factors during this period may have led to changes in academic environments and support systems that reduced reported conflict. Further qualitative research could illuminate these dynamics and help ascertain whether this trend reflects genuine changes in academic pressures or variations in reporting practices among adolescents [46].

**NSSI and Associated Risk Factors.** The NSSI group exhibited significantly elevated rates of depression, PTSD, borderline personality disorder traits, and family conflicts compared to the control group. The association between NSSI and these psychiatric conditions has been well-documented in the literature. For instance, the link between NSSI and conduct disorder reflects the behavioral problems often seen in adolescents who self-harm [47]. Additionally, family conflicts were prevalent among those exhibiting NSSI, corroborating findings by Tatnell et al. (2014), which indicated that interpersonal relationships, particularly within family settings, significantly impacted self-harming behaviors [48]. Also of interest are the results provided by a recently published systematic umbrella review suggesting the potential usefulness of specific interventions targeting emotional dysregulation as a trans-diagnostic manifestation, such as ER Individual Therapy for Adolescents (ERITA), in reducing NSSI behaviors [49].

A particularly salient finding from our analyses is the strong association between a history of self-harm and current NSSI. This underscores the importance of early intervention and preventive efforts targeting adolescents who have previously engaged in self-injurious behavior. Studies, such as those by Ribeiro et al. (2015), have demonstrated that prior self-harm significantly increases the risk of future episodes, reiterating the necessity for routine screenings and interventions in clinical and educational settings to address these behaviors before they escalate [8].

### 4.3. Impact of the COVID-19 Pandemic

The COVID-19 pandemic has generated unprecedented disruptions globally, particularly affecting mental health across various age groups, with adolescents being particularly vulnerable [20,50]. Our study’s comparisons between pre- and post-pandemic periods revealed significant shifts in mental health status and diagnostic profiles among adolescents seeking psychiatric care [51,52]. The findings indicate a complex and multifaceted impact of the pandemic on adolescent mental health, reflecting the interplay of heightened stressors, evolving psychosocial dynamics, and variations in access to mental health services [16,45,53,54].

**Changes in Demographics and Depression Rates.** One of the most striking contrasts observed was the significant increase in the mean age of adolescents in the post-pandemic sample. This demographic shift may suggest various underlying factors, including changes in the age of onset for mental health issues or alterations in treatment-seeking behavior among older adolescents. Previous research has suggested that older adolescents may experience distinct stressors related to transitions into adulthood, which could exacerbate mental health challenges in the context of a pandemic [55].

Moreover, the post-pandemic sample exhibited substantially higher rates of depression and suicidal ideation, aligning with global trends that have documented a surge in these conditions during the pandemic. A meta-analysis by Wang et al. (2022) reported that depression rates among adolescents increased significantly during COVID-19, driven largely by social isolation, uncertainty, and loss [56]. Furthermore, increased family stress and economic instability during the pandemic have been identified as contributing factors to the decline in adolescent mental health [57]. Our findings, which showed statistically significant differences (all *p* < 0.05), underscore the urgency in addressing mental health needs during and following such crises.

**Reports of Potentially Psycho-traumatic Negative Life Events.** In addition to higher rates of depression, the increase in reports of potential psycho-traumatic negative life events among adolescents post-pandemic is concerning. The pandemic has exposed youth to a range of traumatic experiences—ranging from the loss of family members to disruptions in daily life—and these experiences can profoundly impact mental health [58]. Studies have indicated that exposure to such traumatic events significantly heightens the risk of developing PTSD and other stress-related disorders [59]. Furthermore, parental stress has emerged as a significant factor influencing child mental health, underscoring the importance of family dynamics in these contexts [60]. Our findings reaffirm the critical need for mental health services to incorporate trauma-informed approaches in their interventions for adolescents, especially in the context of global crises.

**Decreased Rates of Oppositional Defiant Disorder and Sleep Disorders.** Conversely, we observed lower rates of oppositional defiant disorder (ODD) and sleep disorders in the post-pandemic cohort. This decline might suggest a variety of interpretations, including potential shifts in diagnostic criteria, variations in the contextual factors that contribute to these disorders, or even changes in the environment in which adolescents operate. For instance, with the transition to online learning and decreased traditional school settings, some youth may have experienced reduced external pressures, which could mitigate the expressions of defiant behavior often observed in school contexts [16]. Furthermore, the lower rates of sleep disorders could hint at changes in daily routines and sleep patterns that emerged during lockdowns—such as reduced screen time associated with structured classroom settings and altered family dynamics during this period. However, it is crucial to note that the delayed detection of these disorders may also occur, necessitating the ongoing monitoring of adolescent mental health as the long-term impacts of the pandemic unfold [61].

**Complex Interplay of Stressors and Access to Care.** Overall, these contrasting findings highlight the pandemic’s multifactorial impact on adolescent mental health. They suggest a possible dual effect: while the pandemic has intensified certain stressors (e.g., grief, anxiety, depressive symptoms), it has also altered the context surrounding others, potentially leading to a reduction in some behavior-related disorders. Changes in access to mental health care during this period significantly influenced these outcomes. While some adolescents may have benefited from telehealth services during lockdowns, others encountered barriers, such as technology access, privacy concerns, and reduced in-person support [62]. Implementing online interventions may serve as an effective strategy to support families navigating these challenges [63]. The observed increases in depression and PTSD, alongside demographic shifts and potentially lowered rates of certain disorders, underscore the importance of ongoing surveillance, research, and tailored interventions.

### 4.4. Predictive Models

**Predictive Modeling of Suicidal Behavior.** The present study employed logistic regression models (Models 1 and 2) to evaluate the predictive accuracy for suicidal behavior (SB). These models demonstrated significant predictive accuracy, with Model 1 yielding a χ^2^ statistic of 236.661 (df = 312, *p* < 0.001) and a Tjur R^2^ value of 0.571, while Model 2 offered a slightly improved Tjur R^2^ of 0.599. This level of predictive power suggests that the models effectively identified key variables that contribute to the risk of suicidal behavior in the adolescent population assessed.

Through these models, we identified several significant predictors of SB, including depression, prior suicidal ideation, and, intriguingly, school-related conflicts. Specifically, our findings underscored the substantial risk conferred by depressive symptoms, with Model 1 reporting an odds ratio (OR) of 5.700 (*p* < 0.001) and Model 2 indicating an even greater risk at OR = 6.803 (*p* < 0.001). This aligns with the existing literature that consistently underscores the critical role of depression as a precursor to suicidal behaviors in adolescents [38,39,64].

Moreover, our analyses revealed that a history of suicidal ideation was strongly correlated with current suicidal behaviors, with Model 1 reporting an OR of 47.261 (*p* < 0.001) and Model 2 reflecting an even higher OR of 68.410 (*p* < 0.001). These findings reinforce previous studies that have documented the importance of prior suicidal thoughts as a robust predictor of subsequent suicidal actions, illustrating the urgent need for effective monitoring and intervention strategies for individuals presenting with such histories [8,65]. In this regard, a recently published systematic review indicates that dialectical behavior therapy was the only intervention shown to be effective for adolescents at a high risk for suicide and suicide attempts [66].

Interestingly, our models indicated that school-related conflicts were associated with a decreased risk of SB (Model 1: OR = 0.317, *p* = 0.023). This counterintuitive finding necessitates further investigation, as it diverges from most existing literature that associates academic stress and school-related issues with an increased risk of depression and suicidal behavior [67,68]. Potential explanations for this protective effect could include the role of social support systems within academic settings or variations in coping mechanisms among adolescents facing academic challenges. Further qualitative and quantitative research is warranted to explore the nature of these school-related conflicts, as understanding their impact on suicidal risk could inform targeted interventions within educational frameworks.

**Novel Risk Factor: Parent Living Abroad.** An additional significant finding from Model 2 was the identification of having a parent living abroad as a novel risk factor for suicidal behavior (OR = 11.438, *p* = 0.020). This factor has not been extensively discussed in the literature regarding adolescent suicidal behavior, suggesting a unique area for further exploration. The emotional distance and potential for familial disconnection associated with having a parent reside in a different country could exacerbate feelings of loneliness, abandonment, or instability among adolescents [69,70]. Prior studies have indicated that parental absence or separation can significantly affect a child’s emotional well-being and increase vulnerability to mental health issues [71]. Further research focusing on parenting styles, family dynamics, and the emotional ramifications of having a parent living abroad could elucidate the mechanisms contributing to an increased suicidal risk in this demographic.

**Predictive Modeling of Non-Suicidal Self-Injury (NSSI).** The logistic regression models employed in this study revealed significant predictive capability for non-suicidal self-injury (NSSI), with Model 1 achieving a χ^2^ statistic of 197.244 (df = 312, *p* < 0.001) and a Tjur R^2^ of 0.510, while Model 2 showed an improved Tjur R^2^ of 0.528. These results indicate that our models adeptly identified critical factors contributing to the propensity for NSSI within the adolescent population studied.

**Key Predictors of NSSI.** Among the most striking findings from our analysis was the identification of a history of self-harm as a potent predictor of current NSSI behavior—with Model 1 reporting an odds ratio (OR) of 42.054 (*p* < 0.001) and Model 2 revealing an even greater OR of 52.437 (*p* < 0.001). This finding aligns with a robust body of literature emphasizing the cyclical nature of self-harm behaviors, whereby individuals with a history of self-injury are at a significantly increased risk for future episodes [7,8,72,73]. The persistence of NSSI among adolescents underscores the necessity for vigilant monitoring and effective intervention targeting this population, as early identification and treatment can prevent the escalation of self-harming behaviors. Results of a systematic review indicate some evidence for a reduction in NSSI relapse among adolescents after dialectical behavior therapy (DBT-A) interventions [74].

In addition to the history of self-harm, our models highlighted the significance of comorbidity, with a greater number of comorbid diagnoses emerging as a notable risk factor for NSSI. Specifically, Model 1 indicated an OR of 1.741 (*p* = 0.001), while Model 2 reported an OR of 1.709 (p=0.003). This result resonates with previous research indicating that adolescents with multiple mental health disorders are particularly vulnerable to engaging in self-injurious behaviors [75,76]. Understanding the interplay between diverse comorbidities, such as depression, anxiety, ADHD, and post-traumatic stress disorder (PTSD), is critical for developing comprehensive treatment plans that address the multifactorial nature of mental health challenges in this demographic [77].

**Conduct Disorder and Its Protective Effect.** Interestingly, our findings also revealed that conduct disorder exhibited a protective effect regarding NSSI (Model 1: OR = 0.230, *p* = 0.001; Model 2: OR = 0.184, *p* < 0.001). This unexpected result warrants closer examination, as it diverges from prevailing narratives that associate conduct disorder with higher risks of aggressive or self-injurious behavior among adolescents [78,79]. One possible explanation for this protective association could be that adolescents diagnosed with conduct disorder may express their distress through externalizing behaviors rather than self-harm, thereby reducing the likelihood of engaging in NSSI [80]. However, the relationship may be more nuanced: research suggests that externalizing behaviors and suicidality are indirectly linked, mediated by internalizing factors, and that externalizing behaviors may offer an alternative coping mechanism [80]. Further investigation is necessary to clarify this complex interaction. Alternatively, this finding may reflect underlying differences in coping mechanisms or social support systems within this population, deserving extensive studies to elucidate the complexity of these relationships.

The significant association between comorbidity and NSSI highlights the interconnectedness of various mental health challenges within this population. A comprehensive understanding of these relationships can better inform clinical practice and intervention strategies. For instance, mental health practitioners should conduct thorough assessments of adolescents presenting with self-injurious behavior, including a detailed evaluation of comorbid conditions, to ensure that treatment plans holistically address the patient’s mental health profile [81,82].

**Random Forest Modeling.** While exhibiting lower R^2^ values than logistic regression, the random forest models offer distinct advantages in assessing relative variable importance for both suicidal behavior (SB) and non-suicidal self-injury (NSSI).

For SB, prior suicidal ideation and depression emerged as the most prominent predictors, consistent with the findings from the logistic regression models. However, the random forest approach revealed the influence of additional factors, such as a history of suicide attempts, school-related conflicts, family conflict, and various demographic variables, which may have been obscured by multicollinearity in the logistic regression analyses [83,84]. This ability to uncover hidden relationships within the data underscores the utility of random forest modeling.

Similarly, for NSSI, prior self-harm remained the strongest predictor, affirming the findings of the logistic regression. Notably, the random forest model highlighted the significance of borderline personality disorder traits (BPDs), a factor that was not statistically significant in the logistic regression analysis. This is particularly relevant, as the literature has established the correlation between BPDs and self-injurious behaviors, further emphasizing the need for vigilant screening and assessments in clinical practice [85,86]. The consistent identification of key predictors across both modeling approaches enhances confidence in their clinical relevance and potential implications for intervention strategies.

The observed lower R^2^ values in the random forest models for both outcomes, compared to the logistic regression models, can be attributed to the inherent higher dimensionality of the random forest modeling technique. While logistic regression typically focuses on a selected number of predictor variables, random forest models consider the entire dataset, encompassing a larger number of potential predictors and interactions among them. This complexity, while beneficial for capturing nuanced relationships, can lead to lower R^2^ values as a consequence of how R^2^ is calculated in high-dimensional contexts [87]. It is crucial to note that lower R^2^ values do not imply inferior model performance, particularly regarding the ability of the model to evaluate and rank predictor importance. The implementation of permutation importance in random forest modeling provides a robust method for assessing the contribution of each predictor variable. This approach allows for a comprehensive evaluation of variable importance that can reveal the impact of multiple variables that might otherwise be masked by high collinearity when relying solely on regression coefficients derived from logistic regression [88].

Moreover, the permutation importance method addresses the limitation of traditional significance testing within regression frameworks, where multicollinearity can inflate standard errors and lead to erroneous conclusions about the importance of specific predictors [89]. By revealing hidden relationships and emphasizing the importance of additional factors, like borderline personality disorder traits, the findings from random forest analyses underscore the necessity of utilizing diverse modeling techniques to inform clinical practice and improve outcomes in adolescent mental health.

### 4.5. Limitations

This retrospective cohort study, while offering valuable insights into adolescent self-harm, has several limitations. First, the reliance on existing clinical records introduces recall bias, particularly regarding the self-harm history, trauma, and family history, and poses challenges related to data quality and completeness. Self-reports and parent-reports are prone to memory errors and social desirability bias, where individuals might under- or overreport particular behaviors or experiences. This can lead to inaccurate data, potentially jeopardizing the reliability of our findings. Furthermore, the data may be incomplete due to missing information or varying levels of detail in the original records, which can affect the thoroughness of our analysis. Diagnostic information, based on ICD-10 codes, may not fully capture the complexity of adolescent presentations, leading to the potential underrepresentation of nuanced conditions.

The sample, drawn from a single emergency department, may not be representative of all adolescents who engage in self-harm, as the predominantly female and urban composition limits generalizability. The focus on emergency admissions introduces selection bias, excluding adolescents with less severe presentations who may not seek emergency care. This problem is likely to be particularly reflected when it comes to the composition of our control group, which also includes only patients who presented under emergency conditions. Thus, although it serves our objective of identifying those factors that cause only a proportion of severe psychiatric cases to resort to SB or NSSI, the inclusion of only these cases limits the comparability with non-emergency populations. Furthermore, the broad age range (6-18 years) used in the sample may obscure age-related differences in self-harm behaviors. Measurement error is also a concern, particularly in key variables, like family conflict, trauma, and the socioeconomic status, which were derived from clinical records and may not have been fully documented or consistently reported. Furthermore, another limitation of our study is that specialized questionnaires were not used to quantify the predictor variables, but only a series of predefined questions was used.

The use of both logistic regression and random forest models introduces inherent assumptions, such as the normality of residuals in regression, which may not have been fully met. While multicollinearity was not significant according to Variance Inflation Factors (VIFs), its potential influence on the logistic regression results cannot be ruled out. The subjective nature of hyperparameter selection in random forest models is another limitation, as this may impact model performance and stability.

Moreover, the pre- and post-pandemic comparison does not fully account for other socio-political or economic factors that could have influenced adolescent mental health. Attributing the observed changes solely to the pandemic risks oversimplification, especially when other external variables may have played a role. The specific impact of COVID-19 lockdown measures on access to mental health services, which could have contributed to changes in self-harm behaviors, was not addressed in the study.

The retrospective design prevents the drawing of definitive causal inferences, and observed associations may be influenced by unmeasured confounders. These limitations should be carefully considered when interpreting the findings. Future research utilizing prospective designs and standardized measures is needed to confirm and extend these results.

## 5. Conclusions

This retrospective cohort study examined the risk factors and clinical predictors of suicidal behavior (SB) and non-suicidal self-injury (NSSI) in adolescents presenting to a Romanian pediatric psychiatry emergency department, both before and after the COVID-19 pandemic. The study confirmed a high prevalence of both SB and NSSI among the adolescent sample, consistent with the existing literature. The most common methods of self-harm were cutting and scratching, with a significant increase in prevalence observed post-pandemic. Medication ingestion was the most frequent method for suicide attempts, also showing a notable post-pandemic rise.

Our analysis identified several critical individual, family, and environmental risk factors for both SB and NSSI, including depression, a history of self-harm behaviors, conduct disorder, sleep disorders, anxiety disorders, PTSD, borderline personality traits, family conflict, and exposure to potentially psycho-traumatic negative life events. Both logistic regression and random forest models proved effective in predicting SB and NSSI, with logistic regression offering higher R^2^ values, while random forest provided valuable insights into the relative importance of various predictors. Notably, random forest highlighted the significance of variables, such as a history of suicide attempts and borderline personality traits—factors obscured by multicollinearity in logistic regression models.

The study also revealed significant shifts in adolescent self-harm behaviors following the COVID-19 pandemic, with increases in depression, suicidal ideation, and exposure to possible psycho-traumatic negative life events. These findings align with growing concerns about the pandemic’s detrimental impact on adolescent mental health. Interestingly, we also observed lower rates of family and academic conflict, suggesting a complex interplay of pandemic-related factors that warrant further investigation.

These findings emphasize the need for comprehensive, multidisciplinary approaches to prevent, identify, and treat self-harm behaviors in adolescents. Such interventions should address not only individual-level factors, like depression, anxiety, and other mental health issues, but also the critical role of family and social environments. Early intervention, particularly for adolescents with a history of self-harm, is essential. Further research is needed to better understand the complex and evolving relationships among factors influencing adolescent mental health.

Future studies should focus on prospective longitudinal research to establish definitive causal relationships, expand the sample size to include more diverse populations to improve generalizability, and develop and evaluate culturally sensitive and effective preventive interventions. Additionally, further exploration into how specific pandemic-related factors impacted adolescent mental health and access to care is crucial.

## Figures and Tables

**Table 1:** Main demographic characteristics of the sample and the comparison between the pre-pandemic and post-pandemic periods.

Characteristics	PRE-PANDEMIC (*n* = 194)	POST-PANDEMIC (*n* = 147)	*p*
Female:male distribution (%F)	107:87 (55.15%)	93:54 (63.26%)	0.132 †
Age (M ± SD)	14.289 ± 2.437	14.946 ± 1.922	0.007 *†
Urban:rural provenience (%U)	130:64 (67.01%)	84:63 (57.14%)	0.062 ‡
Family structure (*n*, %)			
Organized	112 (57.73%)	78 (53.06%)	0.390 ‡
Divorced	30 (15.46%)	34 (23.13%)	0.073 ‡
Cohabitation	2 (1.03%)	2 (1.36%)	0.738 ‡
Institutionalized	28 (14.43%)	18 (12.24%)	0.558 ‡
Disorganized (death of one parent)	22 (11.34%)	15 (10.20%)	0.738 ‡
Socioeconomic status (*n*, %)			
Poor	100 (51.54%)	90 (61.22%)	0.075 ‡
Middle	64 (32.98%)	35 (23.81%)	0.064 ‡
Good	28 (14.43%)	18 (12.24%)	0.558 ‡
Very good	2 (1.03%)	4 (2.72%)	0.240 ‡
Conflicts in family (*n*, %)	130 (67.01%)	115 (78.23%)	0.023 *‡
Psychiatric disorders in family (*n*, %)	121 (62.37%)	79 (53.74%)	0.109 ‡
SUD	68 (35.05%)	45 (30.61%)	0.388 ‡
Depression	20 (10.30%)	12 (8.16%)	0.501 ‡
Schizophrenia	14 (7.21%)	12 (8.16%)	0.744 ‡
Anxiety disorders	1 (0.51%)	1 (0.68%)	0.844 ‡
Other disorders	18 (9.27%)	9 (6.12%)	0.285 ‡
Somatic disorders in family (*n*, %)	32 (16.49%)	19 (12.93%)	0.360 ‡
Neurological disorders in family (*n*, %)	2 (1.03%)	6 (4.08%)	0.065 ‡
Potentially traumatic negative life events (*n*, %)			
Death of one parent	22 (11.34%)	15 (10.20%)	0.738 ‡
Death of someone close	8 (4.12%)	6 (4.08%)	0.985 ‡
Divorce or separation of parents	30 (15.46%)	34 (23.13%)	0.073 ‡
Parents abroad	4 (2.06%)	7 (4.76%)	0.162 ‡
Suicide in family	1 (0.51%)	0 (0%)	0.383 ‡
School failure	8 (4.12%)	3 (2.04%)	0.281 ‡
Severe conflicts with friends or parents	24 (12.37%)	31 (21.09%)	0.030 *‡
Serious illness in family or friends	5 (2.58%)	4 (2.72%)	0.935 ‡
Accidents	1 (0.51%)	1 (0.68%)	0.844 ‡
School attendance (*n*, %)	171 (88.14%)	128 (87.07%)	0.766 ‡
School conflicts (*n*, %)	149 (76.80%)	85 (57.82%)	<0.001 *‡
Only with colleagues	99 (51.03%)	51 (34.69%)	0.003 *‡
Only with teachers	10 (5.15%)	6 (4.08%)	0.643 ‡
With both	40 (20.61%)	28 (19.05%)	0.719 ‡
School performance (*n*, %)			0.014 *‡
Poor	121 (62.37%)	89 (60.54%)	0.731 ‡
Middle	53 (27.31%)	30 (20.41%)	0.141 ‡
Good	14 (7.21%)	26 (17.69%)	0.003 *‡
Very good	6 (3.09%)	2 (1.36%)	0.295 ‡
Suicidal behavior (*n*, %)	81 (41.75%)	83 (56.46%)	0.007 *‡
Suicidal ideation (*n*, %)	75 (38.65%)	82 (55.78%)	0.002 *‡
Past suicidal ideation (*n*, %)	86 (44.33%)	77 (52.38%)	0.140 ‡
Suicidal attempt (*n*, %)	39 (20.10%)	42 (28.57%)	0.069 ‡
Past suicidal attempts (*n*, %)	47 (24.23%)	27 (18.37%)	0.194 ‡
Past suicidal behavior (*n*, %)	90 (46.39%)	79 (53.74%)	0.179 ‡
Self-harm (*n*, %)	70 (36.08%)	49 (33.33%)	0.598 ‡
Past self-harm (*n*, %)	83 (42.78%)	75 (51.02%)	0.131 ‡

‡—chi-square; †—*t* test; * = statistical significance.

**Table 2:** Comparison of the distribution of diagnoses and therapeutic interventions between the pre-pandemic and post-pandemic period.

Characteristics	PRE-PANDEMIC (*n* = 194)	POST-PANDEMIC (*n* = 147)	*p*
Diagnostics (*n*)			
Depression	71 (36.60%)	76 (51.70%)	0.038 *‡
CD	74 (38.14%)	61 (41.50%)	0.531 ‡
ADHD	68 (35.05%)	63 (42.86%)	0.142 ‡
ODD	13 (6.70%)	2 (1.36%)	0.017 *‡
Anxiety disorders	58 (29.90%)	43 (29.25%)	0.897 ‡
Sleep disorders	24 (12.37%)	4 (2.72%)	0.001 *‡
SUD	69 (35.57%)	65 (44.22%)	0.105 ‡
Bipolar disorder	6 (3.09%)	4 (2.72%)	0.840 ‡
PTSD	7 (3.61%)	7 (4.76%)	0.595 ‡
Borderline personality disorder	38 (19.59%)	28 (19.05%)	0.901 ‡
No. of comorbid diagnostics (M±SD)	3.077 ± 1.373	3.204 ± 1.385	0.401 †
Psychotherapy (*n*, %)	43 (22.16%)	36 (24.49%)	0.614 ‡
Psychotropic medication (*n*, %)	151 (77.84%)	115 (78.23%)	0.930 ‡

‡—chi-square; †—*t* test; * = statistical significance.

**Table 3:** Main self-harm behaviors before and after the COVID-19 pandemic.

Characteristics	PRE-PANDEMIC	POST-PANDEMIC	*p*
**Self-harm**	***n* = 70**	***n* = 49**	
Cutting, scratching	44 (62.85%)	39 (79.59%)	0.05 *
Self-poisoning	6 (8.57%)	2 (4.08%)	0.121
Other	20 (28.57%)	8 (16.32%)	0.145
**Suicide attempt**	***n* = 39**	***n* = 42**	
Drug ingestion	27 (69.23%)	30 (71.42%)	0.829
Cutting	4 (10.25%)	3 (7.14%)	0.618
Substance intoxication	0 (0%)	1 (2.38%)	0.332
Hanging	0 (0%)	2 (4.76%)	0.168
Defenestration	6 (15.38%)	5 (11.90%)	0.648
Other	2 (5.128%)	1 (2.38%)	0.513

* = statistical significance.

**Table 4:** Characteristics of participants in the main groups.

Characteristics	CONTROL (*n* = 134)	SUICIDAL BEHAVIOR (*n* = 164)			NSSI (*n* = 119)
		Ideation (*n* = 157)	Attempts (*n* = 81)	Whole (*n* = 164)	
Pre-pandemic presentation (*n*, %)	79 (58.95%)	75 (47.77%)	39 (48.15%)	81 (49.39%) ‡	70 (58.82%) ‡
Female:male distribution (%F)	61:73 (45.52%)	114:43 (72.61%)	65:16 (80.24%)	120:44 (73.17%) *‡	79:40 (66.38%) *‡
Age (M ± SD)	14.231 ± 2.678	14.898 ± 1.645	14.864 ± 1.626	14.902 ± 1.670 *†	14.689 ± 1.986 ‡
Urban:rural provenience (%U)	81:53 (60.44%)	104:53 (66.24%)	48:33 (59.25%)	108:56 (65.85%) ‡	79:40 (66.38%) ‡
Family structure (*n*, %)					
Organized	83 (61.94%)	82 (52.23%)	43 (53.09%)	87 (53.05%) ‡	60 (50.42%) ‡
Divorced	21 (15.67%)	39 (24.84%)	20 (24.69%)	39 (23.78%) ‡	20 (16.81%) ‡
Cohabitation	2 (1.49%)	2 (1.27%)	2 (2.47%)	2 (1.22%) ‡	1 (0.84%) ‡
Institutionalized	17 (12.69%)	18 (11.46%)	7 (8.64%)	19 (11.59%) ‡	21 (17.65% )‡
Disorganized (death of one parent)	11 (8.21%)	16 (10.19%)	9 (11.11%)	17 (10.37%) ‡	17 (14.29%) ‡
Socioeconomic status (*n*, %)					
Poor	72 (53.73%)	86 (54.78%)	42 (51.85%)	87 (53.05%) ‡	75 (63.03%) ‡
Middle	40 (29.85%)	45 (28.66%)	24 (29.63%)	49 (29.88%) ‡	29 (24.37%) ‡
Good	21 (15.67%)	21 (13.38%)	12 (14.81%)	23 (14.02%) ‡	11 (9.24%) ‡
Very good	1 (0.75%)	5 (3.18%)	3 (3.70%)	5 (3.05%) ‡	4 (3.36%) ‡
Conflicts in family (*n*, %)	88 (65.67%)	119 (75.80%)	62 (76.54%)	122 (74.39%) ‡	98 (82.35%) *‡
Psychiatric disorders in family (*n*, %)	75 (55.97%)	95 (60.51%)	43 (53.09%)	96 (58.54%) ‡	72 (60.50%) ‡
SUD	41 (30.60%)	51 (32.48%)	25 (30.86%)	51 (31.10%) ‡	45 (37.82%) ‡
Depression	9 (6.72%)	21 (13.38%)	10 (12.35%)	21 (12.80%) ‡	8 (6.72%) ‡
Schizophrenia	10 (7.46%)	12 (7.64%)	5 (6.17%)	12 (7.32%) ‡	10 (8.40%) ‡
Anxiety disorders	1 (0.75%)	1 (0.64%)	0 (0%)	1 (0.61%) ‡	1 (0.84%) ‡
Other disorders	14 (10.45%)	10 (6.37%)	3 (3.70%)	11 (6.71%) ‡	8 (6.72%) ‡
Somatic disorders in family (*n*, %)	20 (14.93%)	22 (14.01%)	13 (16.05%)	24 (14.63%) ‡	20 (16.81%) ‡
Neurological disorders in family (*n*, %)	1 (0.75%)	6 (3.82%)	3 (3.70%)	6 (3.66%) ‡	4 (3.36%) ‡
Potentially traumatic negative life events (*n*, %)					
Death of one parent	11 (8.21%)	16 (10.19%)	9 (11.11%)	17 (10.37%)	17 (14.29%)
Death of someone close	4 (2.99%)	8 (5.10%)	6 (7.41%)	9 (5.49%)	3 (2.52%)
Divorce or separation of parents	21 (15.67%)	39 (24.84%)	20 (24.69%)	39 (23.78%)	20 (16.81%)
Parents abroad	3 (2.24%)	7 (4.46%)	5 (6.17%)	7 (4.27%)	2 (1.68%)
Suicide in family	0 (0%)	0 (0%)	0 (0%)	0 (0%)	1 (0.84%)
School failure	4 (2.99%)	5 (3.18%)	1 (1.23%)	5 (3.05%)	4 (3.36%)
Severe conflicts with friends or parents	15 (11.19%)	29 (18.47%)	20 (24.69%)	31 (18.90%)	23 (19.33%)
Serious illness in family or friends	3 (2.24%)	5 (3.18%)	2 (2.47%)	5 (3.05%)	4 (3.36%)
Accidents	1 (0.75%)	0 (0%)	0 (0%)	0 (0%)	1 (0.84%)
School attendance (*n*, %)	111 (82.84%)	146 (93.00%)	77 (95.06%)	153 (93.29%) *‡	107 (89.92%) ‡
School conflicts (*n*, %)	93 (69.40%)	99 (63.06%)	47 (58.02%)	91 (55.49%) ‡	93 (78.15%) ‡
Only with colleagues	59 (44.03%)	67 (42.68%)	37 (45.68%)	67 (40.85%) ‡	61 (51.26%) ‡
Only with teachers	4 (2.99%)	8 (5.10%)	3 (3.70%)	9 (5.49%) ‡	7 (5.88%) ‡
With both	30 (22.39%)	24 (15.29%)	7 (8.64%)	25 (15.24%) ‡	25 (21.01%) ‡
School performance (*n*, %)					
Poor	86 (64.18%)	86 (54.78%)	41 (50.62%)	88 (53.66%) ‡	73 (61.34%) ‡
Middle	29 (21.64%)	46 (29.30%)	21 (25.93%)	49 (29.88%) ‡	34 (28.57%) ‡
Good	15 (11.19%)	22 (14.01%)	16 (19.75%)	24 (14.63%) ‡	9 (7.56%) ‡
Very good	4 (2.99%)	3 (1.91%)	3 (3.70%)	3 (1.83%) ‡	3 (2.52%) ‡
Suicidal behavior (*n*, %)	0 (0%)	157 (100%)	81 (100%)	164 (100%)	76 (63.87%) ‡
Suicidal ideation	0 (0%)	157 (100%)	74 (91.36%)	157 (95.73%) ‡	75 (63.03%) ‡
Past suicidal ideation	15 (11.19%)	132 (84.08%)	61 (75.31%)	135 (82.32%) *‡	83 (69.75%) ‡
Suicidal attempt	0 (0%)	74 (47.13%)	81 (100%)	81 (49.39%) ‡	40 (33.61%) ‡
Past suicidal attempts	10 (7.46%)	54 (34.39%)	33 (40.74%)	57 (34.76%) *‡	40 (33.61%) ‡
Past suicidal behavior	16 (11.94%)	132 (84.08%)	63 (77.78%)	137 (83.54%) *‡	86 (72.27%) ‡
Self-harm (*n*, %)	0 (0%)	75 (47.77%)	40 (49.38%)	76 (46.34%) ‡	119 (100%)
Past self-harm (*n*, %)	18 (13.43%)	100 (63.69%)	51 (62.96%)	101 (61.59%) *‡	108 (90.76%) ‡

‡—chi-square; †—*t* test; * = statistical significance.

**Table 5:** Comparison of the distribution of diagnoses and therapeutic interventions between the main groups of participants.

Characteristics	CONTROL (*n* = 134)	SUICIDAL BEHAVIOR (*n* = 164)			SELF-HARM (*n* = 119)
		Ideation (*n* = 157)	Attempts (*n* = 81)	Whole (*n* = 164)	
**Diagnostics (*n*, %)**					
Depression	23 (17.16%)	109 (69.43%)	56 (69.14%)	112 (68.29%) *‡	62 (52.10%) *‡
CD	67 (50.00%)	51 (32.48%)	22 (27.16%)	52 (31.71%) *‡	38 (31.93%) *‡
ADHD	56 (41.79%)	50 (31.85%)	26 (32.10%)	53 (32.32%) ‡	48 (40.34%) ‡
ODD	8 (5.97%)	4 (2.55%)	2 (2.47%)	5 (3.05%) ‡	4 (3.36%) ‡
Anxiety disorders	30 (22.39%)	63 (40.13%)	31 (38.27%)	65 (39.63%) *‡	36 (30.25%) ‡
Sleep disorders	15 (11.19%)	5 (3.18%)	3 (3.70%)	5 (3.05%) *‡	12 (10.08%) ‡
SUD	46 (34.33%)	64 (40.76%)	30 (37.04%)	67 (40.85%) ‡	55 (46.22%) ‡
Bipolar disorder	5 (3.73%)	5 (3.18%)	2 (2.47%)	5 (3.05%) ‡	4 (3.36%) ‡
PTSD	0 (0%)	11 (7.01%)	4 (4.94%)	12 (7.32%) *‡	8 (6.72%) *‡
Borderline personality traits	10 (7.46%)	42 (26.75%)	25 (30.86%)	44 (26.83%) *‡	39 (32.77%) *‡
No. of comorbid diagnostics (M ± SD)	2.940 ± 1.255	3.102 ± 1.374	3.111 ± 1.360	3.098 ± 1.376 †	3.546 ± 1.489 *†
Psychotherapy (*n*, %)	23 (17.16%)	45 (28.66%)	18 (22.22%)	45 (27.43%) *‡	38 (31.93%) *‡
Psychotropic medication (*n*, %)	99 (73.88%)	130 (82.80%)	61 (75.31%)	134 (81.71%) ‡	98 (82.35%) ‡

‡—chi-square; †—*t* test; * = statistical significance.

## Data Availability

Data are available on request due to privacy restrictions.

## Supplementary Materials

The following supporting information can be downloaded at: https://www.mdpi.com/article/10.3390/children12010081/s1, Table S1: Predictor variables used in the two regression models.; Table S2: Demographic characteristics of the whole sample of participants.; Table S3: Performance of Model 1 in predicting suicidal behaviors.; Table S4: Performance of Model 2 in predicting suicidal behaviors.; Table S5: Performance of Model 1 in predicting non-suicidal self-injurious behaviors.; Table S6: Performance of Model 2 in predicting non-suicidal self-injurious behaviors.; Table S7: Ranking of all variables used by the random forest model predicting suicidal behavior.; Table S8: Ranking of all variables used by the random forest model predicting NSSI.
