# Supplementary material for: Risk Factors and Clinical Predictors of Suicidal Behaviors and Non-Suicidal Self-Injury Among Pediatric Psychiatry Emergency Admissions Pre- and Post-Pandemic: A Retrospective Cohort Study
Source: Children (Basel). 2025 Jan 11;12(1):81. doi: 10.3390/children12010081 (PMC11764410; doi:10.3390/children12010081)
Supplement: Supplementary file 1 [file children-12-00081-s001.zip › children-3409340-supplementary.pdf]

Table S1. Predictor variables used in the two regression models.

Model 1	Model 2
- Time of presentation: postpandemic = 0; prepandemic = 1<br>- Sex: female = 0; male = 1<br>- Age: continuous variable<br>- Background: rural = 0; urban = 1<br>- Comorbidities (depression, conduct disorder, ADHD, oppositional-defiant disorder, anxiety disorders, sleep disorders, substance use disorder, bipolar affective disorder, PTSD, borderline personality disorder): for each, absence = 0; presence = 1<br>- Number of concurrent psychiatric disorders: continuous variable<br>- Organized family (0/1)<br>- Intra-family conflict (0/1)<br>- Socio-economic status: 1 = low; 2 = medium; 3 = high; 4 = very high<br>- Psychiatric family history (0/1)<br>- Family history of somatic disorders (0/1)<br>- Neurological family history (0/1)<br>- Presence of potential psychotraumatizing events (0/1)<br>- Presence of conflicts in the school environment (0/1)<br>- School results: 1 = poor; 2 = average; 3 = good; 4 = very good	- Subclassifies family structure, each of which is treated dichotomously: organized family, parental divorce, institutionalized child, parental death<br>- Introduces new dimensions related to psychiatric family history, each of the following conditions being coded as 0/1: substance use disorder, depression, schizophrenia, bipolar affective disorder, anxiety disorder, other mental disorders<br>- Subclassifies potential psychotraumatizing events, each coded as 0/1: death of someone close (other than a parent), parents gone abroad, suicide in the family, school failure, severe conflicts with family/friends, serious illness in the family, accidents, other<br>- It foresees several facets of conflicts in the school environment, each coded as 0/1: with peers, with peers and teachers, with teachers only

- Current psychotherapy (0/1)<br>- History of suicidal ideation, suicide attempt or self-harm: each coded 0/1

Table S2. Demographic caracteristics of the whole sample of participants.

Characteristics	WHOLE SAMPLE
Female:male distribution (%F)	200:141 (58.65%)
Age (M±SD)	14.572±2.250
Urban:rural provenience (%U)	214:127 (62.75%)
Diagnostics (n)	
Depression	143
CD	135
ADHD	131
ODD	15
Anxiety disorders	101
Sleep disorders	28
SUD	134
Bipolar disorder	10
PTSD	14
Borderline personality traits	66
No. of comorbid diagnostics (M±SD)	3.132±1.377
Family structure (n)	
Organised	190

Divorced	64
Cohabitation	4
Institutionalised	46
Disorganised (death of one parent)	37
Socioeconomic status (n)	
Poor	190
Middle	99
Good	46
Very good	6
Conflicts in family (n)	245
Psychiatric disorders in family (n)	200
SUD	113
Depression	32
Schizophrenia	26
Anxiety disorders	2
Other disorders	27
Somatic disorders in family (n)	51
Neurological disorders in family (n)	8
Potential psychotraumatising events (n)	
Death of one parent	118
Death of someone close	14
Divorce or separation of parents	64
Parents abroad	11
Suicide in family	1
School failure	11
Conflicts with friends or parents	55
Serious illness in family or friends	9

Accidents	2
School attendance (n)	299
School conflicts (n)	234
Only with colleagues	150
Only with teachers	16
With both	68
School performance (n)	
Poor	210
Middle	83
Good	40
Very good	8
Psychotherapy (n)	79
Psychotropic medication (n)	266
Suicidal behavior (n)	164
Suicidal ideation	157
Suicidal attempt	81
Past suicidal ideation	163
Past suicidal attempts	74
Past suicidal behavior	169
Self-harm (n)	119
Past self-harm (n)	158

Table S3. Performance of Model 1 in predicting suicidal behaviors.

Coefficients

					Wald Test			95% Confidence interval	
	**Estimate**	**Standard Error**	**Odds Ratio**	**z**	**Wald Statistic**	**df**	**p**	**Lower bound**	**Upper bound**
(Intercept)	-1.024	1.785	0.359	-0.574	0.329	1	0.566	-4.521	2.474
Presentation moment	-0.463	0.395	0.629	-1.173	1.376	1	0.241	-1.237	0.310
Gender	-0.275	0.390	0.759	-0.705	0.498	1	0.481	-1.040	0.489
Age	-0.042	0.091	0.959	-0.463	0.215	1	0.643	-0.220	0.136
Provenience	0.144	0.380	1.155	0.380	0.145	1	0.704	-0.600	0.889
Depression	1.740	0.396	5.700	4.392	19.292	1	< .001	0.964	2.517
Conduct disorder	-0.418	0.457	0.659	-0.914	0.836	1	0.361	-1.313	0.478
ADHD	-0.521	0.449	0.594	-1.160	1.346	1	0.246	-1.402	0.359
ODD	-0.260	0.958	0.771	-0.272	0.074	1	0.786	-2.138	1.618
Anxiety	0.188	0.455	1.207	0.413	0.170	1	0.680	-0.704	1.079
Sleep disorders	0.315	0.723	1.371	0.436	0.190	1	0.663	-1.101	1.732
SUD	0.724	0.454	2.062	1.592	2.535	1	0.111	-0.167	1.614
Bipolar disorder	-0.354	0.862	0.702	-0.411	0.169	1	0.681	-2.044	1.335
PTSD	1.040	1.248	2.830	0.834	0.695	1	0.404	-1.405	3.485
Borderline	-0.921	0.527	0.398	-1.747	3.053	1	0.081	-1.955	0.112
No. of comorbidities	-0.025	0.168	0.975	-0.148	0.022	1	0.882	-0.353	0.304
Organized family	-0.143	0.396	0.866	-0.363	0.131	1	0.717	-0.919	0.632
Family conflicts	-0.230	0.489	0.794	-0.471	0.222	1	0.638	-1.188	0.728
SES	-0.021	0.276	0.979	-0.078	0.006	1	0.938	-0.562	0.520
Psychiatric FH	0.324	0.467	1.382	0.693	0.480	1	0.488	-0.592	1.239
Somatic FH	0.416	0.548	1.516	0.759	0.576	1	0.448	-0.658	1.490
Neurological FH	0.562	1.193	1.755	0.472	0.222	1	0.637	-1.775	2.900
Negative events	0.775	0.404	2.171	1.919	3.683	1	0.055	-0.016	1.567
School conflicts	-1.148	0.505	0.317	-2.273	5.165	1	0.023	-2.138	-0.158
School results	0.011	0.251	1.011	0.043	0.002	1	0.966	-0.481	0.503
Psychotherapy	-0.047	0.441	0.954	-0.106	0.011	1	0.916	-0.910	0.817

Coefficients

					Wald Test			95% Confidence interval	
	Estimate	Standard Error	Odds Ratio	z	Wald Statistic	df	p	Lower bound	Upper bound
Past suicidal ideation	3.856	0.503	47.261	7.660	58.673	1	< .001	2.869	4.842
Past self harm	-0.177	0.456	0.838	-0.388	0.151	1	0.698	-1.070	0.717
Past suicidal attempt	-0.173	0.480	0.841	-0.362	0.131	1	0.718	-1.113	0.766

Table S4. Performance of Model 2 in predicting suicidal behaviors

Coefficients

					Wald Test			95% Confidence interval	
	**Estimate**	**Standard Error**	**Odds Ratio**	**z**	**Wald Statistic**	**df**	**p**	**Lower bound**	**Upper bound**
(Intercept)	-1.213	1.833	0.297	-0.661	0.438	1	0.508	-4.805	2.380
Presentation moment	-0.524	0.418	0.592	-1.255	1.576	1	0.209	-1.343	0.294
Gender	-0.338	0.420	0.713	-0.805	0.648	1	0.421	-1.162	0.485
Age	-0.057	0.095	0.945	-0.596	0.355	1	0.551	-0.244	0.130
Provenience	-0.013	0.408	0.987	-0.031	9.543×10^{-4}	1	0.975	-0.812	0.787
Depression	1.917	0.440	6.803	4.354	18.953	1	< .001	1.054	2.780
Conduct disorder	-0.508	0.512	0.602	-0.991	0.982	1	0.322	-1.512	0.496
ADHD	-0.559	0.481	0.571	-1.164	1.355	1	0.244	-1.502	0.383
ODD	-0.023	1.014	0.977	-0.023	5.363×10^{-4}	1	0.982	-2.010	1.963
Anxiety	0.144	0.488	1.155	0.296	0.088	1	0.767	-0.811	1.100
Sleep disorders	0.200	0.774	1.222	0.259	0.067	1	0.796	-1.317	1.718
SUD	0.812	0.497	2.253	1.634	2.669	1	0.102	-0.162	1.786
Bipolar disorder	0.151	0.866	1.163	0.174	0.030	1	0.862	-1.547	1.848
PTSD	1.411	1.668	4.102	0.846	0.716	1	0.397	-1.857	4.680
Borderline	-1.024	0.566	0.359	-1.809	3.272	1	0.070	-2.134	0.086
No. of comorbidities	-0.021	0.179	0.979	-0.117	0.014	1	0.907	-0.371	0.330
Family conflicts	-0.101	0.528	0.904	-0.191	0.037	1	0.848	-1.135	0.933
SES	0.089	0.290	1.093	0.306	0.093	1	0.760	-0.479	0.656
Somatic FH	0.643	0.569	1.902	1.129	1.276	1	0.259	-0.473	1.759
Neurological FH	1.196	1.310	3.308	0.913	0.834	1	0.361	-1.371	3.764
School results	0.044	0.266	1.045	0.164	0.027	1	0.869	-0.478	0.565
Psychotherapy	-0.483	0.495	0.617	-0.975	0.951	1	0.330	-1.454	0.488
Past suicidal ideation	4.226	0.582	68.410	7.265	52.774	1	< .001	3.085	5.366
Past self harm	-0.162	0.497	0.850	-0.326	0.106	1	0.744	-1.136	0.812
Past suicidal attempt	-0.054	0.533	0.947	-0.101	0.010	1	0.919	-1.098	0.990
Parental divorce	0.939	0.533	2.557	1.763	3.108	1	0.078	-0.105	1.983

Coefficients

					Wald Test			95% Confidence interval	
	Estimate	Standard Error	Odds Ratio	z	Wald Statistic	df	p	Lower bound	Upper bound
Institutionalized	0.959	0.672	2.610	1.427	2.036	1	0.154	-0.358	2.277
Parental death	-0.171	0.634	0.843	-0.269	0.072	1	0.788	-1.413	1.072
FH SUD	0.198	0.557	1.219	0.355	0.126	1	0.722	-0.894	1.289
FH Depression	1.309	0.838	3.703	1.563	2.443	1	0.118	-0.333	2.951
FH Schizophrenia	-0.058	0.822	0.943	-0.071	0.005	1	0.943	-1.670	1.554
FH Anxiety	0.958	2.448	2.606	0.391	0.153	1	0.696	-3.840	5.755
FH Other psychiatric disorders	0.623	0.877	1.864	0.710	0.504	1	0.478	-1.097	2.342
Death of someone close	-0.050	0.868	0.951	-0.058	0.003	1	0.954	-1.752	1.652
Parents abroad	2.437	1.049	11.438	2.324	5.401	1	0.020	0.382	4.492
Suicide in family	-17.152	1455.398	3.558×10^{-8}	-0.012	1.389×10^{-4}	1	0.991	-2869.679	2835.376
School failure	0.649	1.050	1.913	0.618	0.381	1	0.537	-1.410	2.707
Severe conflicts	1.091	0.590	2.976	1.847	3.413	1	0.065	-0.066	2.248
Serious illness	0.592	1.063	1.807	0.557	0.310	1	0.578	-1.492	2.676
Accidents	-12.904	995.602	2.489×10^{-6}	-0.013	1.680×10^{-4}	1	0.990	-1964.248	1938.441
Conflicts w/ colleagues	-1.125	0.553	0.325	-2.032	4.129	1	0.042	-2.209	-0.040
Conflicts w/ teachers	-2.130	0.943	0.119	-2.258	5.100	1	0.024	-3.979	-0.281
Conflicts w/ both	-1.797	0.714	0.166	-2.519	6.346	1	0.012	-3.196	-0.399

Table S5. Performance of Model 1 in predicting non-suicidal self-injurious behaviors.

Coefficients

					Wald Test			95% Confidence interval	
	**Estimate**	**Standard Error**	**Odds Ratio**	**z**	**Wald Statistic**	**df**	**p**	**Lower bound**	**Upper bound**
(Intercept)	-4.614	1.752	0.010	-2.634	6.936	1	0.008	-8.048	-1.180
Presentation moment	0.632	0.393	1.881	1.608	2.587	1	0.108	-0.138	1.401
Gender	-0.186	0.403	0.831	-0.460	0.212	1	0.645	-0.976	0.604
Age	-0.079	0.085	0.924	-0.920	0.846	1	0.358	-0.246	0.089
Provenience	0.350	0.373	1.419	0.938	0.880	1	0.348	-0.381	1.082
Depression	-0.009	0.423	0.991	-0.021	4.395×10^{-4}	1	0.983	-0.838	0.820
Conduct disorder	-1.468	0.448	0.230	-3.280	10.757	1	0.001	-2.345	-0.591
ADHD	-0.088	0.438	0.916	-0.201	0.040	1	0.841	-0.946	0.770
ODD	-1.406	0.920	0.245	-1.528	2.335	1	0.126	-3.209	0.397
Anxiety	-0.763	0.465	0.466	-1.642	2.696	1	0.101	-1.675	0.148
Sleep disorders	0.384	0.697	1.468	0.551	0.303	1	0.582	-0.982	1.750
SUD	0.131	0.412	1.140	0.317	0.101	1	0.751	-0.678	0.939
Bipolar disorder	-0.812	0.980	0.444	-0.828	0.686	1	0.408	-2.734	1.110
PTSD	-0.378	0.802	0.685	-0.471	0.222	1	0.637	-1.949	1.193
Borderline	0.012	0.431	1.012	0.028	8.109×10^{-4}	1	0.977	-0.833	0.858
No. of comorbidities	0.554	0.174	1.741	3.183	10.130	1	0.001	0.213	0.896
Organized family	0.115	0.396	1.122	0.289	0.084	1	0.772	-0.662	0.892
Family conflicts	0.543	0.522	1.722	1.042	1.085	1	0.297	-0.479	1.565
SES	0.211	0.265	1.235	0.796	0.633	1	0.426	-0.308	0.730
Psychiatric FH	-0.159	0.447	0.853	-0.356	0.126	1	0.722	-1.036	0.717
Somatic FH	0.784	0.570	2.191	1.375	1.890	1	0.169	-0.334	1.902
Neurological FH	0.839	1.049	2.313	0.799	0.639	1	0.424	-1.217	2.895
Negative events	0.113	0.363	1.119	0.310	0.096	1	0.757	-0.600	0.825
School conflicts	0.506	0.491	1.658	1.029	1.058	1	0.304	-0.458	1.469

Coefficients

					Wald Test			95% Confidence interval	
	Estimate	Standard Error	Odds Ratio	z	Wald Statistic	df	p	Lower bound	Upper bound
School results	0.058	0.257	1.060	0.226	0.051	1	0.822	-0.445	0.561
Psychotherapy	-0.455	0.400	0.635	-1.137	1.292	1	0.256	-1.239	0.329
Past suicidal ideation	0.265	0.471	1.303	0.561	0.315	1	0.574	-0.659	1.188
Past self harm	3.739	0.480	42.054	7.786	60.620	1	< .001	2.798	4.680
Past suicidal attempt	0.043	0.447	1.044	0.096	0.009	1	0.923	-0.833	0.919

Table S6. Performance of Model 2 in predicting non-suicidal self-injurious behaviors

Coefficients

					Wald Test			95% Confidence interval	
	Estimate	Standard Error	Odds Ratio	z	Wald Statistic	df	p	Lower bound	Upper bound
(Intercept)	-3.796	1.781	0.022	-2.132	4.545	1	0.033	-7.286	-0.306
Presentation moment	0.620	0.406	1.858	1.528	2.334	1	0.127	-0.175	1.415
Gender	-0.246	0.432	0.782	-0.570	0.325	1	0.568	-1.093	0.600
Age	-0.124	0.090	0.884	-1.380	1.904	1	0.168	-0.299	0.052
Provenience	0.284	0.410	1.329	0.694	0.482	1	0.487	-0.518	1.087
Depression	0.122	0.450	1.130	0.271	0.073	1	0.786	-0.759	1.003
Conduct disorder	-1.691	0.485	0.184	-3.490	12.179	1	< .001	-2.641	-0.741
ADHD	0.078	0.485	1.081	0.161	0.026	1	0.872	-0.872	1.028
ODD	-1.535	1.043	0.215	-1.471	2.165	1	0.141	-3.579	0.510
Anxiety	-0.896	0.495	0.408	-1.812	3.283	1	0.070	-1.866	0.073
Sleep disorders	0.520	0.756	1.682	0.687	0.473	1	0.492	-0.962	2.002
SUD	0.217	0.435	1.243	0.499	0.249	1	0.618	-0.636	1.071
Bipolar disorder	-1.203	1.068	0.300	-1.127	1.270	1	0.260	-3.295	0.889
PTSD	-0.463	0.835	0.629	-0.555	0.308	1	0.579	-2.101	1.174
Borderline	0.100	0.450	1.105	0.223	0.050	1	0.824	-0.782	0.982
No. of comorbidities	0.536	0.182	1.709	2.938	8.631	1	0.003	0.178	0.894

Coefficients

					Wald Test			95% Confidence interval	
	Estimate	Standard Error	Odds Ratio	z	Wald Statistic	df	p	Lower bound	Upper bound
Family conflicts	0.371	0.556	1.449	0.667	0.445	1	0.505	-0.718	1.460
SES	0.217	0.287	1.242	0.754	0.569	1	0.451	-0.346	0.780
Somatic FH	0.779	0.591	2.180	1.319	1.740	1	0.187	-0.379	1.937
Neurological FH	0.835	1.122	2.305	0.745	0.554	1	0.456	-1.363	3.034
School results	0.036	0.274	1.037	0.132	0.018	1	0.895	-0.501	0.574
Psychotherapy	-0.449	0.434	0.638	-1.034	1.070	1	0.301	-1.299	0.402
Past suicidal ideation	0.229	0.509	1.258	0.451	0.203	1	0.652	-0.768	1.227
Past self harm	3.960	0.526	52.437	7.533	56.753	1	< .001	2.929	4.990
Past suicidal attempt	0.086	0.482	1.090	0.179	0.032	1	0.858	-0.858	1.030
Parental divorce	-0.349	0.548	0.706	-0.637	0.406	1	0.524	-1.422	0.724
Institutionalized	0.172	0.650	1.188	0.265	0.070	1	0.791	-1.102	1.447
Parental death	0.338	0.622	1.402	0.544	0.296	1	0.586	-0.880	1.557
FH SUD	-0.265	0.532	0.767	-0.497	0.247	1	0.619	-1.308	0.779
FH Depression	-0.763	0.695	0.466	-1.097	1.203	1	0.273	-2.126	0.600
FH Schizophrenia	0.438	0.789	1.549	0.555	0.308	1	0.579	-1.109	1.984
FH Anxiety	1.427	2.181	4.166	0.654	0.428	1	0.513	-2.848	5.702
FH Other psychiatric disorders	0.395	0.823	1.484	0.479	0.230	1	0.632	-1.219	2.009
Death of someone close	-0.114	0.927	0.892	-0.123	0.015	1	0.902	-1.931	1.703
Parents abroad	-0.028	1.148	0.972	-0.024	5.910×10^{-4}	1	0.981	-2.277	2.221
Suicide in family	13.749	882.744	935413.688	0.016	2.426×10^{-4}	1	0.988	-1716.397	1743.895
School failure	0.408	1.040	1.503	0.392	0.154	1	0.695	-1.631	2.447
Severe conflicts	0.461	0.538	1.586	0.858	0.736	1	0.391	-0.593	1.516
Serious illness	0.940	1.224	2.561	0.768	0.590	1	0.442	-1.459	3.340
Accidents	-0.223	1.998	0.800	-0.112	0.012	1	0.911	-4.139	3.693
Conflicts w/ colleagues	0.273	0.538	1.314	0.507	0.257	1	0.612	-0.781	1.327
Conflicts w/ teachers	1.489	0.872	4.431	1.707	2.914	1	0.088	-0.220	3.198
Conflicts w/ both	0.454	0.667	1.574	0.680	0.463	1	0.496	-0.853	1.760

Table S7. Ranking of all variables used by the random forest model predicting suicidal behavior.

Impurity importance		Permutation importance	
1.	Past_suicidal_ideation: 16.7	1.	Past_suicidal_ideation: 0.129
2.	Depression: 7.66	2.	Depression: 0.0390
3.	Age: 2.45	3.	Past_suicidal_attempt: 0.00931
4.	Past_suicidal_attempt: 2.06	4.	Gender_F1: 0.00591
5.	Past_NSSI: 1.92	5.	Past_NSSI: 0.00572
6.	No_comorbidities: 1.84	6.	Age: 0.00287
7.	Gender_F1: 1.74	7.	Anxiety: 0.00251
8.	School_results: 1.26	8.	ADHD: 0.00249
9.	SES: 1.14	9.	Prepandemic_1: 0.00234
10.	Anxiety: 1.03	10.	Severe_conflicts: 0.00190
11.	Prepandemic_1: 0.918	11.	Conflicts_both: 0.00179
12.	ADHD: 0.893	12.	Sleep_disorders: 0.00177
13.	Severe_conflicts: 0.876	13.	Divorce: 0.00173
14.	Provenience_U1: 0.811	14.	SES: 0.00165
15.	FH_SUD: 0.780	15.	CD: 0.00146
16.	CD: 0.738	16.	School_results: 0.00141
17.	Divorce: 0.729	17.	Provenience_U1: 0.00125
18.	Psychointerventions: 0.654	18.	FH_SUD: 0.000898
19.	Conflicts_colleagues: 0.642	19.	PTSD: 0.000765
20.	Conflicts_both: 0.629	20.	No_comorbidities: 0.000603
21.	SUD: 0.612	21.	FH_Neurological: 0.000360
22.	Borderline: 0.559	22.	SUD: 0.000311
23.	Treatment: 0.558	23.	Conflicts_colleagues: 0.000277
24.	Parental_death: 0.539	24.	Psychointerventions: 0.000270
25.	FH_Somatic: 0.512	25.	Family_conflicts: 0.000180

26.	Sleep_disorders: 0.495	26.	Serious_illness: 0.000100
27.	Conflicts_teachers: 0.442	27.	Borderline: 0.0000543
28.	Family_conflicts: 0.439	28.	Accidents: 0.0000507
29.	Institutionalized: 0.353	29.	Bipolar: 0.0000507
30.	Bipolar: 0.327	30.	Treatment: 0.0000301
31.	FH_Depression: 0.322	31.	FH_Bipolar: 0
32.	Family_suicide: 0.310	32.	FH_Anxiety: 0
33.	PTSD: 0.295	33.	Family_suicide: 0
34.	FH_Neurological: 0.287	34.	FH_Depression: -0.000130
35.	Serious_illness: 0.270	35.	Parental_death: -0.000211
36.	FH_Schizophrenia: 0.267	36.	FH_Schizophrenia: -0.000228
37.	ODD: 0.230	37.	School_failure: -0.000297
38.	School_failure: 0.229	38.	Parents_abroad: -0.000332
39.	Parents_abroad: 0.221	39.	ODD: -0.000369
40.	FH_Others: 0.202	40.	Close_death: -0.000371
41.	Close_death: 0.160	41.	FH_Somatic: -0.000517
42.	Accidents: 0.0811	42.	Conflicts_teachers: -0.000557
43.	FH_Anxiety: 0.0319	43.	FH_Others: -0.000699
44.	FH_Bipolar: 0	44.	Institutionalized: -0.000873

Table S8. Ranking of all variables used by the random forest model predicting NSSI.

Impurity importance		Permutation importance	
1.	Past_NSSI: 16.9	1.	Past_NSSI: 0.136
2.	No_comorbidities: 3.14	2.	Borderline: 0.0107
3.	Past_suicidal_ideation: 2.42	3.	No_comorbidities: 0.00895
4.	Age: 2.25	4.	Past_suicidal_ideation: 0.00877
5.	Borderline: 1.83	5.	School_results: 0.00569
6.	School_results: 1.74	6.	CD: 0.00544
7.	SES: 1.24	7.	Anxiety: 0.00282
8.	Prepandemic_1: 1.05	8.	Prepandemic_1: 0.00175
9.	Past_suicidal_attempt: 1.05	9.	Conflicts_colleagues: 0.00158
10.	CD: 0.901	10.	Past_suicidal_attempt: 0.00150
11.	Psychointerventions: 0.889	11.	SES: 0.00142
12.	Anxiety: 0.813	12.	Family_conflicts: 0.00115
13.	Conflicts_colleagues: 0.758	13.	Psychointerventions: 0.00106
14.	Gender_F1: 0.724	14.	Gender_F1: 0.000928
15.	Family_conflicts: 0.684	15.	PTSD: 0.000889
16.	Provenience_U1: 0.681	16.	Depression: 0.000789
17.	Treatment: 0.639	17.	Parents_abroad: 0.000518
18.	FH_SUD: 0.634	18.	ADHD: 0.000504
19.	Depression: 0.633	19.	Conflicts_both: 0.000503
20.	ADHD: 0.589	20.	Parental_death: 0.000455
21.	Parental_death: 0.570	21.	Sleep_disorders: 0.000200
22.	SUD: 0.564	22.	FH_SUD: 0.000155
23.	Severe_conflicts: 0.542	23.	Conflicts_teachers: 0.000154
24.	FH_Somatic: 0.519	24.	FH_Bipolar: 0
25.	Divorce: 0.483	25.	Family_suicide: 0

26.	Parents_abroad: 0.475	26.	FH_Anxiety: -0.0000142
27.	Institutionalized: 0.471	27.	FH_Others: -0.0000163
28.	Conflicts_both: 0.417	28.	FH_Neurological: -0.0000378
29.	Conflicts_teachers: 0.409	29.	FH_Somatic: -0.0000527
30.	FH_Depression: 0.397	30.	Bipolar: -0.0000870
31.	PTSD: 0.366	31.	Accidents: -0.000105
32.	Sleep_disorders: 0.361	32.	Close_death: -0.000113
33.	FH_Others: 0.340	33.	ODD: -0.000193
34.	FH_Schizophrenia: 0.331	34.	SUD: -0.000202
35.	Bipolar: 0.244	35.	FH_Depression: -0.000208
36.	ODD: 0.228	36.	Divorce: -0.000221
37.	Serious_illness: 0.193	37.	Age: -0.000288
38.	Close_death: 0.190	38.	Provenience_U1: -0.000315
39.	School_failure: 0.173	39.	School_failure: -0.000353
40.	FH_Neurological: 0.170	40.	Serious_illness: -0.000382
41.	Accidents: 0.108	41.	FH_Schizophrenia: -0.000413
42.	Family_suicide: 0.0333	42.	Institutionalized: -0.000783
43.	FH_Anxiety: 0.0314	43.	Severe_conflicts: -0.000882
44.	FH_Bipolar: 0	44.	Treatment: -0.000971
